# Evidence for the requirement of 14-3-3eta (YWHAH) in meiotic spindle assembly during mouse oocyte maturation

**DOI:** 10.1186/1471-213X-13-10

**Published:** 2013-04-01

**Authors:** Santanu De, Douglas Kline

**Affiliations:** 1 Department of Biological Sciences, Kent State University, Kent, OH, 44242, USA

**Keywords:** Meiosis, Oocyte maturation, 14-3-3η, Meiotic spindle, Morpholino oligonucleotide, α-tubulin

## Abstract

**Background:**

The 14-3-3 (YWHA) proteins are central mediators in various cellular signaling pathways regulating development and growth, including cell cycle regulation. We previously reported that all seven mammalian 14-3-3 isoforms are expressed in mouse oocytes and eggs and that, 14-3-3η (YWHAH) accumulates and co-localizes in the region of meiotic spindle in mouse eggs matured *in vivo*. Therefore, we investigated the role of 14-3-3η in spindle formation during mouse oocyte maturation.

**Results:**

Examination of oocytes matured *in vitro* demonstrated that 14-3-3η accumulates in both meiosis I and II spindles. To explore if 14-3-3η interacts directly with α-tubulin in meiotic spindles, we performed an *in situ* proximity ligation assay that can detect intracellular protein-protein interactions at the single molecule level and which allows visualization of the actual interaction sites. This assay revealed a marked interaction between 14-3-3η and α-tubulin at the metaphase II spindle. To demonstrate a functional role for 14-3-3η in oocyte maturation, mouse oocytes were microinjected with a translation-blocking morpholino oligonucleotide against 14-3-3η mRNA to reduce 14-3-3η protein synthesis during oocyte maturation. Meiotic spindles in those cells were examined by immunofluorescence staining of 14-3-3η and α-tubulin along with observation of DNA. In 76% of cells injected with the morpholino, meiotic spindles were found to be deformed or absent and there was reduced or no accumulation of 14-3-3η in the spindle region. Those cells contained clumped chromosomes, with no polar body formation. Immunofluorescence staining of 14-3-3η and α-tubulin in control eggs matured *in vitro* from uninjected oocytes and oocytes microinjected with the ineffective, inverted form of a morpholino against 14-3-3η, a morpholino against 14-3-3γ, or deionized water showed normal, bipolar spindles.

**Conclusions:**

The results indicate that 14-3-3η is essential for normal meiotic spindle formation during *in vitro* maturation of mouse oocytes, in part by interacting with α-tubulin, to regulate the assembly of microtubules. These data add to our understanding of the roles of 14-3-3 proteins in mouse oocyte maturation and mammalian reproduction.

## Background

In female mammals, meiosis is initiated prenatally and oocytes remain arrested for long periods in an immature state at prophase of the first meiotic division. Within the ovary, the oocyte is held in arrest by signals from the surrounding granulosa cells. This arrest is released as a result of the preovulatory surge in luteinizing hormone mediated by the granulosa cells and activation of maturation promoting factor (MPF) within the oocyte. MPF triggers the resumption of meiosis leading to a second arrest at metaphase II forming the mature, fertilizable egg [1-4]. Failure of oocyte maturation has been documented in animal models and must be considered when human female infertility is examined [5].

Central to this process of oocyte maturation is the correct formation of the metaphase spindles that ensure the accurate alignment and separation of chromosomes at each meiotic division [6-10]. Centrioles are absent in female mouse oocytes and spindle assembly during the two meiotic divisions is therefore independent of centrioles [11]. Examination of spindle assembly in live mouse oocytes revealed that the spindle assembles from many microtubule organizing centers (MTOCs) that are present in the prophase oocyte and that increase in number after nuclear envelope breakdown [12]. It is clear that centrioles are absent in mouse oocytes and the term “acentriolar centrosomes” has been used to describe the MTOCs that ultimately form the spindle; however other researchers refer to mouse oocytes as acentrosomal to denote the formation of a centrosomal equivalent from a clustering of MTOCs [reviewed in 9]. Regardless of the terminology used, the centrosome or MTOCs (centrosomal equivalent) material associated with the MTOCs contain proteins associated with centrosomes in mitotic cells, including γ-tubulin, pericentrin, Nuclear Mitotic Apparatus (NuMA) protein and other proteins [reviewed in 9]. Aurora kinase A (AURKA) appears to be central in regulating MTOC number during oocyte maturation [13] and microtubule motor proteins aid in the formation and elongation of the bipolar spindle [12].

During oocyte maturation, haploid eggs are produced by two successive divisions that are asymmetric to produce a large egg cell and smaller polar bodies. This process involves both the assembly of the bipolar spindle coordinated by chromatin and spindle positioning at the periphery, which is dependent on the interaction between actin and chromatin. Translocation of the spindle and chromosomes to the periphery also promotes differentiation of the cortex producing a microvilli-free and actin-rich zone that characterizes the site of polar body formation [14,15].

Proteins of the 14-3-3 (YWHA) family are now known to be central mediators in a variety of cellular signaling pathways involved in development and growth including cell cycle regulation and apoptosis [16-20]. There is strong evidence from studies of the cell cycle in somatic cells that 14-3-3 is involved in signaling systems regulating cell division [21,22]. There is also recently reported evidence from studies of *Dictyostelium* that 14-3-3 coordinates the interaction between the mitotic spindle and cytokinesis [23,24] as well as some evidence that 14-3-3 is associated with the mitotic apparatus in mammalian cells [25]. Thus, there is some indication that 14-3-3 proteins have a role in spindle and cytoskeleton function; however, the role of 14-3-3 proteins in mouse meiotic spindle formation and function is unknown. We previously found that all seven mammalian isoforms of 14-3-3 are expressed in mouse ovaries, oocytes and eggs and showed that 14-3-3η accumulates and co-localizes with α-tubulin in the region of the meiotic spindle in mouse eggs matured *in vivo*[26] suggesting that 14-3-3η has a functional role.

In the work presented here, we performed an *in situ* proximity ligation assay (PLA) to determine if 14-3-3η interacts directly with α-tubulin in the meiotic spindle. The PLA has been used successfully to not only detect protein-protein interactions at the single molecule level directly in cells, but also to visualize the actual intracellular sites of the interactions in different types of cells and tissues [27-29]. In the PLA method, specific primary antibodies (raised in different species) bind to target proteins. A pair of oligonucleotide-conjugated secondary antibodies (PLA probes) bind to the primary antibodies and when the PLA probes are in close proximity (<40 nm), the DNA strands are joined by enzymatic ligation. A circular DNA molecule is generated and then amplified by rolling circle amplification. The original *in situ* protein-protein interaction is revealed by the amplified DNA detected with a fluorescent probe. The PLA technique is sensitive, specific, and provides a high signal to noise ratio because the signal is amplified and close proximity of the target proteins is required. Thus, the method permits detection of two proteins that interact at the molecular level.

To begin an investigation of the role of 14-3-3η in spindle formation, we performed experiments to reduce the 14-3-3η protein in mouse oocytes by interfering with translation of the 14-3-3η message. A number of techniques that rely on reducing protein expression by RNA interference have been effective in identifying key protein functions in oocytes, eggs and early embryos of mice and other species. These techniques include RNAi-mediated methods, including RNAi transgenic approaches [30-34]; however, we chose to study the role of 14-3-3η in meiotic spindle formation during oocyte maturation, by reducing the synthesis of 14-3-3η protein by intracellular microinjection of a translation-blocking morpholino oligonucleotide against 14-3-3η.

Morpholino oligomers are small sequences of synthetic nucleotides consisting of about 25 standard nucleic acid bases attached to morpholine rings (rather than ribose rings) with a phosphorodiamidate non-ionic linkage instead of a phoshodiester linkage (giving the oligonucleotide a net neutral charge) [35]. While a number of methods have been developed to block gene expression at the level of the mRNA, including those using the ability of cellular RNAase H-dependent methods and siRNA techniques, the development of morpholinos to knock down proteins by blocking translation has overcome a number of limitations associated with the other techniques. With the appropriate experimental controls [36], morpholinos have a number of advantages including specificity resistance to nucleases. Morpholinos appear to have few off-target interactions and little non-antisense activity because they are specific (binding to at least 13–14 contiguous bases) and the neutral charge gives little interaction with other RNA species or cellular proteins [37,38]. The feasibility of using morpholino oligomers to block a target gene’s function in mouse has been established through a morpholino phenocopy of the easily discernible and well-characterized mouse mos−/− phenotype [39]. Morpholino oligonucleotides have been successfully used to inhibit translation of specific genes in embryos of zebrafish [40] and *Xenopus*[41]. Similarly, experiments utilizing morpholino antisense oligomers have implicated a number of proteins in mouse oocyte meiotic maturation [39,42-50].

In this study, using several methods, we show that 14-3-3η interacts with α-tubulin especially in the meiotic spindles in mouse oocytes and eggs. Moreover, using morpholino-mediated knockdown of 14-3-3η, we provide evidence that 14-3-3η is required for normal meiotic spindle assembly during most oocyte maturation.

## Results and discussion

### The protein 14-3-3η co-localizes with α-tubulin at the metaphase II spindles in mouse eggs

**Figure 1:**
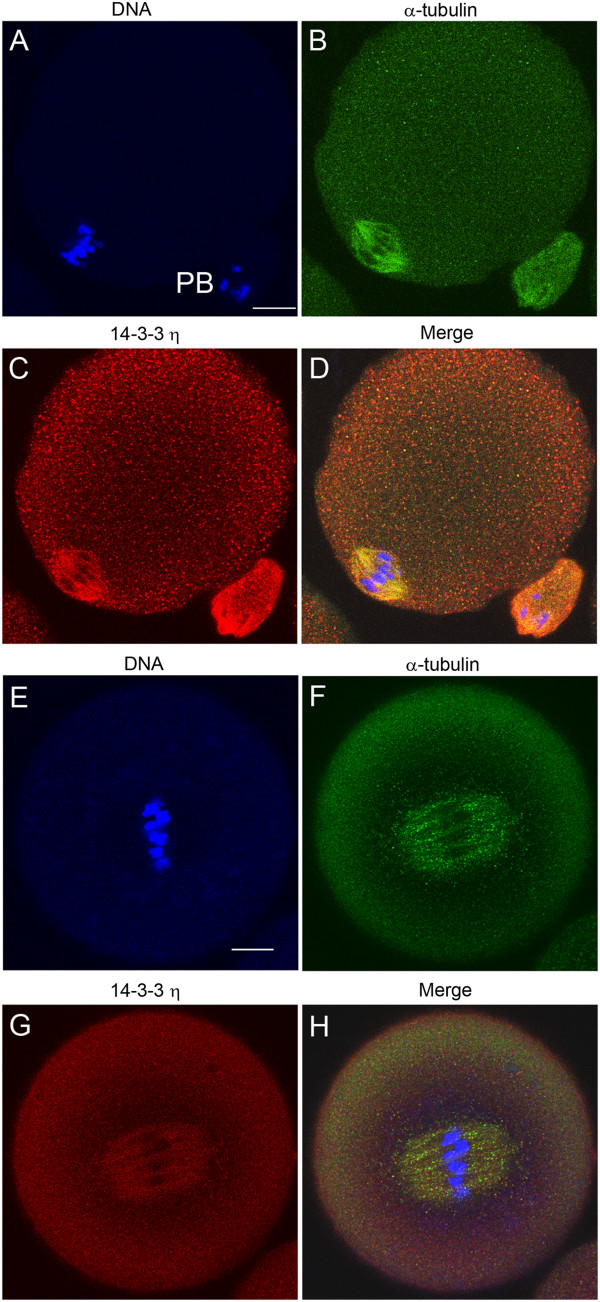
**The 14-3-3η protein accumulates at the metaphase II spindle of the mouse egg matured *in vivo and in vitro*.** Cells were fixed, permeabilized and immunolabeled for confocal double immunofluorescence using one of two primary antibodies against the 14-3-3η protein (red), an antibody to α-tubulin (green) and counterstained with Hoechst 33342 (blue) to visualize DNA. (**A-D**) A representative *in vivo*-matured and ovulated egg labeled with a goat antibody that recognizes the C-terminal end of the 14-3-3η protein (**C**). (**E-H**) A representative *in vitro*-matured egg cell that was held in prophase I arrest for 24 hours, released from the arrest and examined at 13 hours with a rabbit antibody recognizing the N-terminal end of the 14-3-3η protein (**G**). **PB**, First Polar Body. The merged image is an overlay of immunofluorescence images from the three channels. Scale bars represent 10 μm.

We previously demonstrated that 14-3-3η is uniformly distributed in fully grown, immature mouse oocytes with greater distribution in the cytoplasm than in germinal vesicles, and that it accumulates at the metaphase II spindle of eggs matured *in vivo*[26]. In that study, indirect immunofluorescence, utilizing a rabbit antibody directed against the cross-reacting N-terminal sequence of sheep 14-3-3η, demonstrated that 14-3-3η, but none of the other 14-3-3 isoforms, is concentrated at the metaphase II spindle and that 14-3-3η appeared to co-localize with α-tubulin. In the present study, we have confirmed this observation using an entirely different goat-derived antibody generated against a peptide mapping near the C-terminus of 14-3-3η of human origin. We found again, in all 20 *in vivo*-matured and ovulated eggs examined, evidence for the accumulation of 14-3-3η at the metaphase II spindle. As shown in Figure 1A-D, this antibody confirms the co-localization of 14-3-3η with α-tubulin in the metaphase spindle of *in vivo*-matured eggs as well as with α-tubulin in the first polar body.

There are some differences in the shape and size of the meiotic spindle during *in vivo* maturation compared to *in vitro* maturation, for example, it has been reported that *in vivo*-matured eggs have pointed spindles with compact localization of γ-tubulin at the spindle poles. In contrast *in vitro*-matured eggs exhibited large more barrel shaped spindles with γ-tubulin distributed over more spindle microtubules [51]. These observations, however, do not represent fundamental differences in spindle function in as much as chromosomes segregate and polar bodies form in both cases. As our studies here rely on *in vitro* maturation, we first confirmed that 14-3-3η accumulates in the spindle of *in vitro* matured eggs using the rabbit antibody used in the previous study of mature ovulated eggs.

We detected prominent accumulation of 14-3-3η at the spindle region in 20 of 23 in eggs matured *in vitro* (Figure 1E-H). These cells, in anticipation of the experiments to follow, were held as oocytes in an arrested state at prophase I for 24 hours with 0.1 mg/ml dibutyryl cAMP (dbcAMP) in the media. After 24 hours the dbcAMP was removed to allow maturation, and the cells were examined about 13 hours later, a time at which mature eggs are formed. We used the rabbit antibody targeted against the N-terminal sequence of 14-3-3η exclusively for the remaining experiments in this report.

### The protein 14-3-3η accumulates and co-localizes with α-tubulin at both meiosis I and II spindles during *in vitro* mouse oocyte maturation

**Figure 2:**
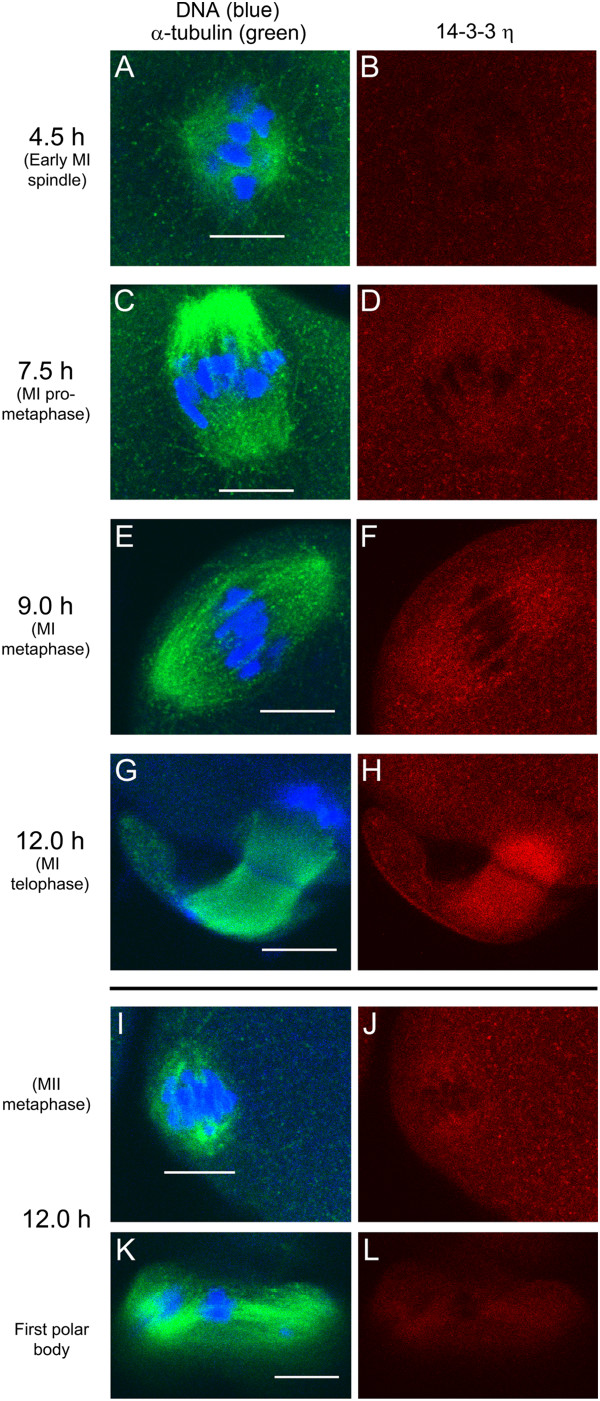
**The 14-3-3η protein accumulates and co-localizes with α-tubulin in MI and MII meiotic spindles during oocyte maturation.** Representative cells were fixed, permeabilized and immunolabeled for confocal microscopy during oocyte *in vitro* maturation at the times indicated. The left column shows the merged images of α-tubulin (green) and the counterstain Hoechst 33342 (blue) to visualize the DNA. The right column shows each corresponding image of the 14-3-3η protein (red). (**A-H**) Paired confocal images at a single confocal plane of the spindle region of representative cells at the times indicated. (**I-L)** Representative egg imaged at the plane of metaphase II spindle (**I,J**) and at a different confocal plane to show the first polar body (**K,L**) attached to the same egg cell. Scale bars represent 10 μm.

Given that 14-3-3η is concentrated at the metaphase II spindle of mouse eggs, we examined the co-localization of 14-3-3η with α-tubulin during the process of *in vitro* oocyte maturation by additional confocal indirect immunofluorescence studies. Oocytes were collected in HEPES-buffered MEM α containing dbcAMP, and then allowed to mature *in vitro* by incubation in bicarbonate-buffered MEM α without dbcAMP for 4.5 hours (early spindle formation), 7.5 hours (MI pro-metaphase), 9 hours (MI metaphase) and 12 hours (MI telophase to MII metaphase) before fixation [6]. We examined 15 cells at each stage of maturation and found the following pattern in all cells examined. The 14-3-3η protein was found to be present throughout the cytoplasm during oocyte maturation at all stages (Figure 2A-J). Accumulation of 14-3-3η at the meiotic spindle region was detected around prometaphase I at 7.5 hours after release from prophase I arrest (Figure 2C-D). By 9 hours of maturation, a marked co-localization of 14-3-3η with α-tubulin was observed at the metaphase I spindle with condensed chromosomes aligned at the mid-spindle region (Figure 2E-F). At telophase I, prominent accumulation of 14-3-3η was found at the broad midbody microtubules during formation of the first polar body (Figure 2G-H). At the end of 12 hours of the maturation, telophase I is followed quickly by the formation of the metaphase II spindle (Figure 2I) and again 14-3-3η was shown to accumulate in the region of the MII spindle (Figure 2I-J) as well as associated with α-tubulin in the first polar body (Figure 2K-L).

These experiments suggest a functional role of a specific isoform of 14-3-3 proteins, 14-3-3η, in the formation of normal meiotic spindles during mouse oocyte maturation. The marked accumulation of 14-3-3η at the metaphase II meiotic spindles in eggs was observed by immunofluorescence staining of the protein using two individual primary antibodies raised against different immunogens, one against the N-terminus and the other against the C-terminus of the peptide sequence. The gradual accumulation and co-localization of 14-3-3η at the meiosis I and II spindles observed during mouse oocyte maturation *in vitro*, suggest that the 14-3-3η protein may be involved with concomitant formation of the spindles by directly or indirectly influencing the assembly of the spindle microtubules.

The 14-3-3 proteins are known to bind to a large number other proteins to regulate many cellular processes [16,19]; however, the role of 14-3-3 proteins in development of the mammalian meiotic spindle has not yet been examined. Some studies have suggested that isoforms of 14-3-3 are associated with tubulin and may play a role in microtubule assembly and spindle formation in mitosis. Proteomic analysis of interphase and mitotic HeLa cells demonstrated that 14-3-3 proteins interact with α- and β-tubulin in both interphase and mitotic cells [20,52,53]. The 14-3-3γ and 14-3-3ε proteins have been reported to be localized in the centrosomes and mitotic spindle of mouse leukemic FDCP cells and at least one isoform is associated with the centrosomes and spindle of mouse 3T3 cells [25]. The 14-3-3 protein has also been found to interact with ENDOSPERM DEFECTIVE 1 (EDE1), a plant microtubule-associated protein essential for plant cell division and for microtubule organization in endosperm [54]. We have further explored the potential interaction of 14-3-3η with α-tubulin by extending the indirect immunofluorescence co-localization studies to examine the direct interaction of 14-3-3η with α-tubulin at the molecular level, as described below.

### Evidence for direct association of 14-3-3η with α-tubulin and accumulation of the interactions at the metaphase II spindle

**Figure 3:**
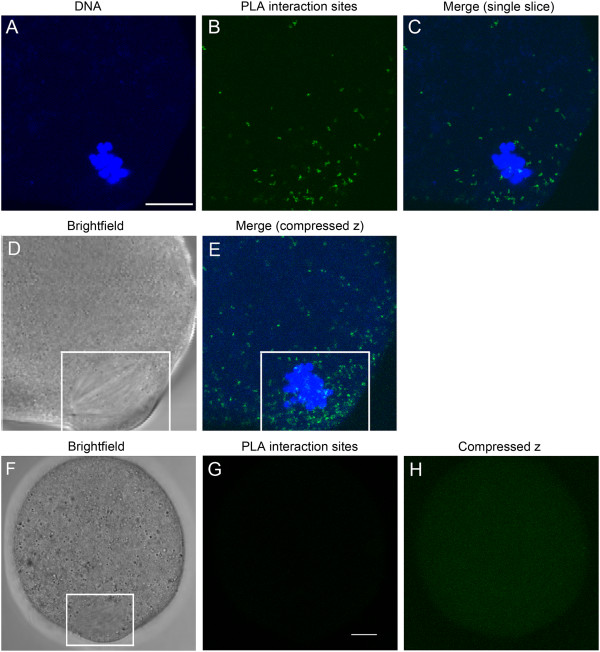
**The 14-3-3η protein interacts directly with α-tubulin.** (**A-C**) A single confocal section through the region of the meiotic spindle of a representative *in vitro*-matured egg. Sites of protein 14-3-3η and α-tubulin are indicated by the green fluorescent spots (**B**). The cell was counterstained with Hoechst 33342 (blue) to visualize DNA (**A**) and the merged image is shown (**C**). (**D**) The non-confocal, brightfield image of this cell and spindle (white box). (**E)** A compressed stack of seven consecutive confocal scans performed at 2 μm intervals from the bottom of the spindle to its top. A marked accumulation of the *in situ* PLA sites of interaction of 14-3-3η with α-tubulin was observed at the meiotic spindles in eggs and along the egg cortices about the spindles (highlighted by the white box). (**F-H**) A representative control egg processed simultaneously for *in situ* PLA by identical procedure, in absence of the primary antibodies for 14-3-3η and α-tubulin. (**F**) The non-confocal, brightfield image of the cell and its spindle (white box). (**G**) A single, confocal fluorescence scan through the spindle region of the egg cell showing no *in situ* PLA fluorescent reaction spot. (**H**) A compressed stack of all confocal scans from the bottom of the egg cell to its top, showing complete absence of *in situ* PLA fluorescent reaction spots. Background fluorescence was minimal (**G, H**). Scale bars represent 10 μm.

The observation that 14-3-3η co-localizes with α-tubulin in the double labeling immunofluorescence experiments does not necessarily mean that the 14-3-3η protein is interacting with α-tubulin directly. We examined protein-protein interactions at the single molecule level using the *in situ* proximity ligation assay (PLA) and documented the distribution of intracellular sites of the interactions between 14-3-3η and α-tubulin in mouse eggs matured *in vitro.* The *in situ* PLA revealed some interactions (noted by distinct bright fluorescent reaction spots) of 14-3-3η with α-tubulin throughout the cytoplasm of all mouse eggs matured *in vitro*, along with a prominent accumulation of the interaction sites at the meiotic spindles (Figure 3A-E) in 16 out of 18 eggs examined. In addition, an abundance of the interaction sites was noted along the cell cortices next to the spindles of those eggs studied (Figure 3C,E). These observations indicate that 14-3-3η interacts with α-tubulin in mouse eggs and that such interactions are dramatically more prevalent in the region of the meiotic spindle as would be predicted by the co-localization and enhanced concentration indicated by the immunofluorescence experiments.

No *in situ* PLA fluorescent reaction spots were detected throughout control mouse eggs processed simultaneously following the identical procedure but without addition of the primary antibodies (Figure 3F-H). In the absence of primary antibodies there is no ligation and rolling circle amplification of the PLA probes that is necessary for detection. In addition, no background fluorescence from unhybridized probes is detected in confocal sections imaged at the same confocal setting as the experimental cells (Figure 3G) and only a very slight background is apparent in the compressed Z images (Figure 3H).

We do not yet know if 14-3-3η and tubulin form part of a larger macromolecular complex. Additional studies may indicate if 14-3-3η also interacts with other proteins at the spindle and elsewhere in the cell. The 14-3-3 protein forms functional homodimers or heterodimers of different isoforms [55,56]. In some cases, the separate isoforms may have overlapping functions and may be exchangeable in binding to a target protein either as homodimers of isoforms or heterodimers of mixed isoforms. In other cases there may be a specific isoform preference for interaction with some target proteins [57]. We found no evidence for accumulation of any of the other six 14-3-3 isoforms at the meiotic spindle in mature mouse eggs [26], suggesting that the interaction we observed is mediated by 14-3-3η homodimers, and that other isoforms are not specifically associated with the spindle. Moreover, as shown by the following experiments, a reduction in 14-3-3η protein alone causes defects in spindles, indicating that there is no functional overlap with the other 14-3-3 isoforms.

### A 14-3-3η translation-blocking morpholino causes absence or deformation of the meiosis I spindle during *in vitro* mouse oocyte maturation

The results described above clearly demonstrated that 14-3-3η is closely associated with α-tubulin during the formation of meiosis I and II and spindles. To begin to understand the functional role of 14-3-3η in the formation of meiotic spindles during mouse oocyte maturation, we did a series of experiments in which the amount of 14-3-3η protein in the oocyte was effectively reduced by inhibiting translation of the 14-3-3η message. While in prophase I arrest, GV-intact oocytes were microinjected with a translation-blocking morpholino oligonucleotide against 14-3-3η at a final intracellular concentration of 0.1 mM and held for 24 hours in prophase arrest to permit a reduction of the existing14-3-3η protein. The oocytes were then released from the meiotic arrest, allowed to mature *in vitro,* fixed at 13 hours after the release from meiotic arrest and examined by confocal indirect immuno-fluorescence.

**Figure 4:**
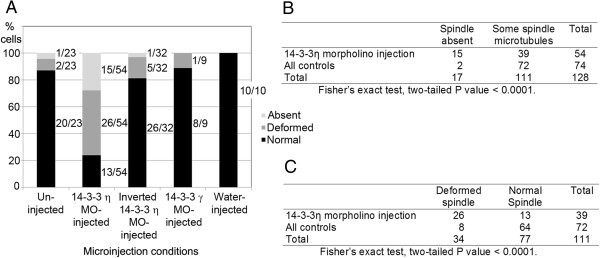
**Summary of experimental results on meiotic spindle structure following injection of the 14-3-3η morpholino and control conditions.** As described in Methods, oocytes were held in prophase I meiotic arrest for 24 hours, allowed to mature *in vitro* for 13 hours and then processed for immunofluorescence and confocal microscopy to examine the spindle structure. **(A)** For each condition, the number of cells with a normal spindle, deformed spindle or no spindle is represented graphically as a percentage of the total number of cells examined. Light grey, spindle absent; dark grey, deformed spindle; black, normal spindle. The number of cells with absent, deformed or normal spindles out of the total number of cells studied for each injection condition is indicated alongside the corresponding percentage bars. Representative images of cells displaying the three categories are presented in Figure 5 and 6. (**B**) Fisher’s Exact test comparing the experimental group (14-3-3η morpholino injection) and combined controls for the absence of spindles or presence of at least some spindle. (**C**) Fisher’s Exact test comparing the experimental group (14-3-3η morpholino injection) and combined controls for deformed or normal spindle. MO, morpholino oligonucleotide.

**Figure 5:**
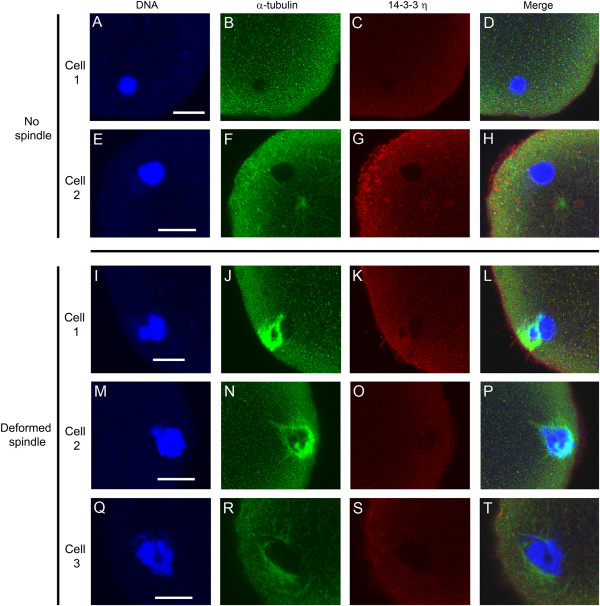
**Microinjection of a morpholino against 14-3-3η causes absence or deformation of meiotic spindles in cells matured *in vitro*.** Oocytes were injected with 0.1 mM 14-3-3η morpholino, held for 24 hours in prophase I arrest, released from the arrest for 13 hours, fixed, permeabilized and immunolabeled for confocal immunofluorescence with an antibody to α-tubulin (green), the antibody to 14-3-3η protein (red), and counterstained with Hoechst 33342 (blue) to visualize the DNA. The panel on the far right is the merged overlay of immunofluorescence images from all three channels. (**A-D** and **E-H**) The upper two rows (cells 1 and 2) are images of two representative cells that have clumped DNA, no spindle and no 14-3-3η accumulation. (**I-L**, **M-P**, and **Q-T**) The lower 3 rows (cells 1–3) are representative images of cells that have deformed spindles, disorganized DNA and no accumulation of 14-3-3η at the spindle region. None of these cells injected with the 14-3-3η morpholino formed a first polar body, indicating that the disrupted spindles shown here are MI spindles. Scale bars represent 10 μm.

Cells underwent germinal vesicle breakdown, but reduction of 14-3-3η protein caused a substantial decrease in the number of cells that formed a normal bipolar spindle during first meiosis. Only 24% of the cells injected with the morpholino oligonucleotide targeting 14-3-3η formed an apparently normal bipolar spindle, while in 76% of the cells the spindle was absent or deformed with no polar body formation (Figure 4A). Immunofluorescence images of two representative morpholino-injected cells that formed no spindle are shown in Figure 5A-H. It can be seen that, in these cases, the DNA is clumped (Figure 5A,E), the spindle microtubules are absent (Figure 5B,F), and there is no accumulation of 14-3-3η around the chromatin or in the region where the spindle should be forming (Figure 5C,G). The spindle regions of three representative cells with deformed meiotic spindle are shown in Figure 5I-T. In these cases, the DNA is partially clumped (Figure 5I,M,Q) and the spindle is unipolar, disorganized, or apolar and incompletely formed (Figure 5J,N,R). Again, there is little or no accumulation of 14-3-3η detected in these incompletely formed spindle regions (Figure 5K,O,S). None of these cells injected with the 14-3-3η morpholino formed polar bodies, indicating that such cells, with absent or deformed meiosis I spindles, do not progress to cytokinesis and first polar body formation.

As indicated by indirect immunofluorescence, some 14-3-3η protein was detected throughout the cytoplasm of eggs matured from oocytes microinjected with the morpholino against 14-3-3η mRNA (Figure 5C,G,K,O,S). This may be residual protein produced before the morpholino injection as well as some protein translated from 14-3-3η mRNA not completely blocked by the morpholino. It is not possible to make a quantitative comparison of 14-3-3η protein content using fluorescence microscopy because, while controls were imaged along with the experimental cells, there will be small inherent differences in primary and secondary antibody concentration and binding and the confocal optics may be slightly different. Clearly however, though some of the protein might be present in the cytoplasm, the absence of 14-3-3η accumulation at the meiotic spindle is striking, indicating a reduction in the protein that is targeted to the spindle. Moreover, the reduction in the 14-3-3η protein around the DNA correlates with the absence or abnormal formation of the spindle.

There will be some variability in the 14-3-3η protein concentration and knock-down effects. The amount of 14-3-3η protein may have been sufficient to permit spindle formation in the 24% of 14-3-3η morpholino-injected cells we categorized as having a spindle that appeared normal. We observed polar bodies associated with about half (7/13) of these cells indicating the cells proceeded to metaphase II. Other cells had no adhering polar body. This suggests that these cells formed an apparently normal metaphase I spindle, but did not continue with cytokinesis, which could be the case. However, the polar body sometimes breaks within the *zona pellucida* surrounding mature eggs and the polar body often becomes dissociated when *zona*-free eggs are pipetted through processing media drops, so that by the time these cells are examined carefully with confocal microscopy the polar body of a mature metaphase II egg may be absent. Therefore the small percentage of cells with a normal spindle may have proceeded to metaphase II or stopped at metaphase I. In any event, the 14-3-3η morpholino injection prevents spindle formation or caused abnormal spindles in over 76% of cells injected. The limitation in the knockdown procedure to completely eliminate the 14-3-3η protein can be overcome by gene knockout experiments and we are currently developing a conditional 14-3-3η knockout mouse.

**Figure 6:**
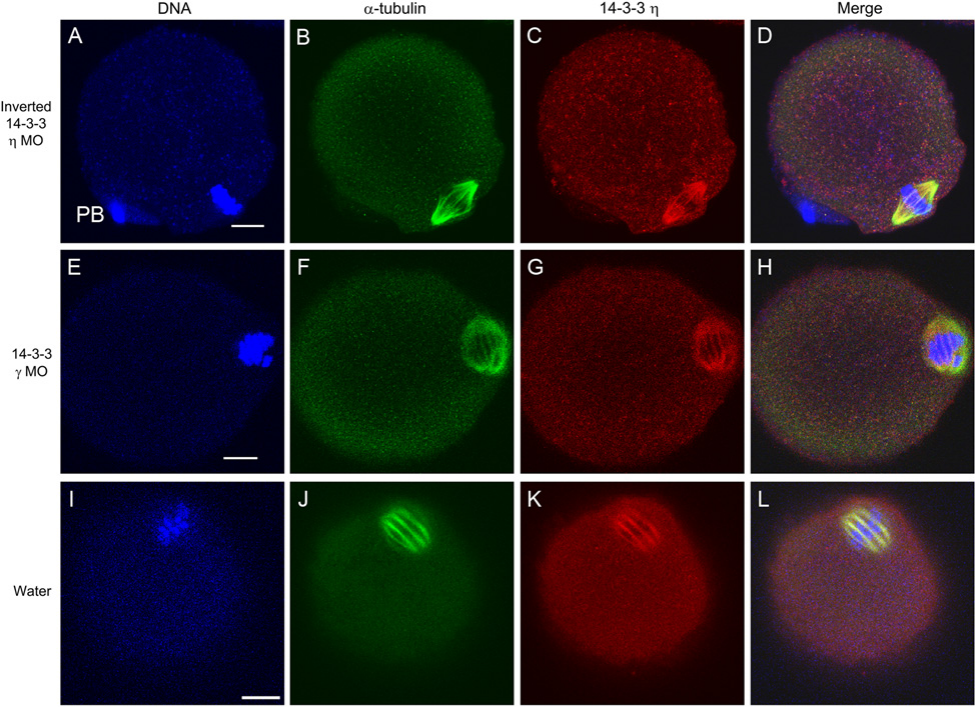
**Representative control eggs matured *in vitro *from injected oocytes, showing normal, bipolar meiotic spindles.** (**A-D**) Images of an oocyte injected with 0.01 mM of the inverted 14-3-3η morpholino. (**E-H**) Images of an oocyte injected with 0.01 mM the 14-3-3γ morpholino. (**I-L**) Images of an oocyte injected with 10 pL of deionized water. In all cases these oocytes, representative of others (see Figure 4) both injected and uninjected, were held for 24 hours in prophase arrest, released from arrest for13 hours, fixed, permeabilized and immunolabeled for confocal immunofluorescence with an antibody to α-tubulin (green), the antibody to 14-3-3η protein (red), and counterstained with Hoechst 3342 (blue) to visualize DNA. The panel at the far right is the merged overlay of immunofluorescence images from all three channels. In all cases, the spindle appears normal and 14-3-3η accumulates at the spindle. PB, first polar body. MO, morpholino oligonucleotide. Scale bars represent 10 μm.

For comparison with the 54 experimental cells injected with the morpholino against 14-3-3η, we examined a total of 51 eggs matured *in vitro* from oocytes injected with several control solutions. Like the experimental cells, these oocytes were injected, held in arrest for 24 hours and then allowed to mature. We also examined 23 uninjected cells treated in the same manner. The results are summarized in Figure 4 and representative images are shown in Figure 6. For the series of injected control cells, 86% had normal metaphase II spindles and, for the uninjected cells, 87% had normal metaphase II spindles (compared to only 24% of cells injected with the 14-3-3η morpholino, as indicated before).

One series of control experiments utilized the inverted form of the morpholino oligonucleotide against 14-3-3η mRNA; a panel of immunofluorescence images of a representative egg cell is shown in Figure 6A-D. The inverted morpholino cannot bind to the 14-3-3η mRNA and should not knockdown protein synthesis. In such cells, the metaphase II spindle formed, 14-3-3η accumulated at the metaphase II spindle and the cell completed first polar body formation. We also injected some cells with a translation-blocking morpholino targeting 14-3-3γ (YWHAG). This 14-3-3 isoform is known to be present in the mouse oocyte and mature egg, though it does not accumulate in the egg spindle region [26]. Injection of an equivalent amount of this morpholino did not disrupt spindle formation nor seemed to affect oocyte maturation; a representative egg cell is shown in Figure 6E-H. Finally, since the morpholinos were dissolved in deionized water, some mouse oocytes were microinjected with an equivalent amount of deionized water alone. When treated in the same manner as the experimental cells, these cells formed normal metaphase II spindles (Figure 6I-L).

Statistical analysis of 2×2 contingency tables using Fisher’s Exact test validated the significant differences between the experimental and control groups (Figure 4B,C). Pairwise comparison across the four control groups revealed no significant difference among them; as a result, all control groups were combined. When the absence of spindles is compared to the presence of at least some spindle microtubules between the experimental group and combined controls, the two-tailed P value is less than 0.0001. When the presence of deformed spindles is compared to the presence of normal spindles between the experimental group and the combined controls, the two-tailed P value is less than 0.0001. The analysis reveals a very strong statistical difference between the 14-3-3η morpholino injection group and controls when the number of absent spindles is scored (Figure 4B) and a very strong statistical difference between the 14-3-3η morpholino injection group and controls when the number of deformed spindles is scored (Figure 4C). Each analysis indicates that injection of 14-3-3η morpholino blocks or disrupts spindle formation.

The results of microinjection of the translation-blocking morpholino oligonucleotide against 14-3-3η confirms that this particular isoform of 14-3-3 is essential for meiosis I spindle formation. Except for some cells in which the 14-3-3η morpholino may have been less effective, the majority of cells form no spindle or a deformed spindle when examined 13 hours after release from meiotic arrest. In those cases, the spindle and chromosomes do not resemble any of the transition stages we would expect to see if the spindle formation were merely delayed. Based on live cell imaging of spindle formation in mouse eggs, the spindle forms from a collection and expansion of microtubule organizing centers that aggregate in a ball just after germinal vesicle breakdown. A bipolar spindle forms with progressive clustering of MTOCs and activity of motor proteins. During this process**,** individual condensed bivalent chromosomes form during the initial contacts with microtubules. Thus the chromosomes remain individualized throughout the process of spindle formation [12]. The absent or deformed spindles we observe in 14-3-3η morpholino-injected cells do not represent transitional states. Aggregating MTOCs and individualized condensed chromosomes should be present continuously after germinal vesicle breakdown. Instead, the 14-3-3η morpholino injections show that spindle formation is prevented and that the DNA forms a clump in the absence of a spindle or if the spindle is abnormally formed. When maturing oocytes are treated with colchicine [14] or nocodazole [12] to depolymerize microtubules, the spindle doesn’t form and the chromosomes collapse into a single mass very similar to the appearance of the DNA that we see in oocytes injected with 14-3-3η morpholino. Interestingly, one or several chromosome masses move to the cortex in oocytes treated with colchicine during oocyte maturation [14] as we also observed in our experiments (Figure 5A-H). Thus our results are somewhat analogous to the inhibition of spindle formation by microtubule depolymerizing agents, but we do see in some cases a partial, imperfect spindle associated with a chromatin mass of indistinct chromosomes (Figure 5I-T). These results suggest that 14-3-3η is required at a sufficient concentration to enable the aggregation of MTOCs to form a complete bipolar spindle.

In the absence of spindle formation and first polar body formation, the oocyte would not proceed to metaphase II. We do not yet know if reducing 14-3-3η protein alters other cytoplasmic maturation events that would affect the ability of the cell to be fertilized or develop a polyspermy block characteristic of a normal metaphase II-arrested egg. Nor do we know if more subtle changes in 14-3-3 activity and spindle structure might be associated with aneuploidy. There may be some changes in the spindles of 14-3-3η morpholino-injected cells in which we see an apparently normal looking spindle that could lead to aneuploidy or other developmental defects after fertilization. It is known for mammalian eggs that maintenance of spindle integrity is important in preventing aneuploidy and that aneuploidy in meiosis I is common and increases with maternal age in humans [reviewed in 9,10].

With the morpholino-mediated knockdown of the 14-3-3η isoform, other isoforms, though present, do not appear to substitute or compensate for the absence of 14-3-3η, indicating that 14-3-3η is the central 14-3-3 protein required for normal spindle formation. The 14-3-3 proteins have been implicated in controlling mitosis in somatic cells; however the exact correspondence of regulatory mechanisms by 14-3-3 in somatic cells or oocytes of other species with the mouse oocyte will need to be explored further in the light of our findings. For example, in somatic (HeLa) cells, 14-3-3η depletion appears to disrupt chromosome segregation and disrupt mitosis, leading to cell death. The identity of the 14-3-3η target proteins in these cells was not examined, but depletion of 14-3-3η also sensitizes the cells to several microtubule inhibitors, suggesting an interaction with tubulin [58].

Our results indicate a specific interaction of 14-3-3η with α-tubulin in the meiotic spindle and that this interaction is required for normal spindle formation. It may be that 14-3-3η interacts with other proteins individually or in a complex with tubulin. In addition to playing a role in spindle formation, 14-3-3η could also function in other maturation events apart from a direct interaction with tubulin and formation of the spindle. The 14-3-3 proteins are known to be important in regulating mitosis through interactions with CDC25 (cell division cycle 25) proteins [22,59-62] and some evidence implicates 14-3-3 proteins in serving to maintain meiotic arrest at prophase I in mouse oocytes [63,64]. The experiments reported here indicate that reduction of 14-3-3η or 14-3-3γ protein synthesis by morpholino injection, under the conditions we have used, does not interfere with the resumption of meiosis following the removal of dbcAMP since oocyte nuclear envelope breakdown occurs normally. We are currently investigating which of the 14-3-3 proteins is involved in the regulation of cell cycle control proteins, particularly CDC25B (cell division cycle 25B) phosphatase.

The 14-3-3 proteins have also been found to regulate cell mechanics and cytokinesis in somatic cells by integrating key cytoskeletal components. In *Dictyostelium*, for example, where only one 14-3-3 isoform is known to be present, partial knockdown of 14-3-3 protein does not appear to alter spindle formation during mitosis, though it does cause cytokinesis defects resulting in multi-nucleated cells [23]. Similar mechanical events in meiosis would suggest a role for 14-3-3 as well [65]. It has been suggested that the central spindle found in anaphase of animal cells during mitosis is required for formation of the contractile ring. A number of microtubule-bundling and stabilizing factors are required for formation of the central spindle; among them is centralspindlin which is regulated by Aurora B and 14-3-3. In this case, 14-3-3 apparently acts to sequester centralspindlin, maintaining it in an inactivate state until it is phosphorylated by Aurora B, and is released from 14-3-3 to form centralspindlin clusters in the central spindle [66]. The characteristic asymmetric cell division in meiosis apparently requires different organization and regulation though, for example, the Aurora kinases are involved with the regulation of cytokinesis in oocytes [67,68]. Additional studies may reveal possible interactions of 14-3-3 with target proteins associated with cytokinesis.

## Conclusions

We have shown, for the first time, the functional importance of a specific isoform of 14-3-3, namely 14-3-3η, in regulating meiotic spindle formation during mouse oocyte maturation *in vitro*, by morpholino-mediated knock-down of the protein. The results of the study indicate that 14-3-3η regulates the organization or stabilization of meiosis I spindle assembly and may be required for first polar body formation, thereby allowing normal progression to metaphase II spindle formation as well. *In situ* proximity ligation assays and confocal indirect immunofluorescence experiments demonstrate that 14-3-3η interacts with α-tubulin in eggs, with an accumulation of the interactions at the meiotic spindle. These results suggest that 14-3-3η is essential for formation of the normal meiotic spindle apparatus during mouse oocyte maturation *in vitro* by interacting, in part with α-tubulin to regulate the assembly of microtubules.

## Methods

### Collection of oocytes and eggs

All mice were housed and used at Kent State University under an approved Institutional Animal Care and Use Committee protocol following the National Research Council’s publication Guide for the Care and Use of Laboratory Animals. Oocytes and eggs were collected as previously described [69]. Adult (2–3 months old) CF1 mice were injected with 7.5 IU eCG (G4877, Sigma) and, 44–48 hours later, the ovaries were removed and repeatedly punctured with a 26-gauge needle to rupture follicles. Cumulus cell-enclosed oocytes were isolated and the cumulus cells were removed by repeated pipetting though a small-bore pipette. Fully-grown oocytes with intact nuclei (germinal vesicles) were collected and cultured in Minimum Essential Medium α modification (MEM α) (12000–014, Invitrogen) containing 120 U/ml penicillin G (P4687, Sigma), 50 μg/ml streptomycin sulfate (S1277, Sigma), 0.24 mM sodium pyruvate (P-4562, Sigma), 0.1% polyvinyl alcohol (P-8136, Sigma), and buffered with 20 mM HEPES to pH 7.2. Throughout these experiments with oocytes, except where indicated, 0.1 mg/ml dibutyryl cAMP (D0627, Sigma) was added to prevent spontaneous oocyte maturation. Mature, metaphase-II arrested eggs were obtained from adult CF1 mice 13 hours after injection of 7.5 IU hCG (CG10, Sigma) which was preceded by a priming injection of 7.5 IU eCG injection 48 hours earlier. Cumulus cells were removed with 0.3 mg/ml hyaluronidase (H4272, Sigma). Cumulus-free eggs were collected in MEM α.

### Immunofluorescence and confocal microscopy

Cells were briefly treated in acid Tyrode’s solution solution (0.14 M NaCl, 3 mM KCl, 1.6 mM CaCl_2_.2H_2_O, 0.5 mM MgCl_2_.6H_2_O, 5.5 mM glucose, and 0.1% PVA, pH 2.5) to remove *zonae pellucidae*. Cells were then fixed in freshly prepared 3.7% paraformaldehyde for 30–60 minutes, washed in 0.1% PVA in PBS, permeabilized with 1% triton X-100 (X100, Sigma), washed in 0.1% PVA in PBS and in blocking buffer containing 5% normal donkey serum (017-000-121, Jackson Immunoresearch), and incubated at 4°C overnight simultaneously with rabbit anti-14-3-3η (AHP1046, AbD Serotec) and rat anti-α-tubulin (sc-69970, Santa Cruz Biotechnology), each diluted 1:200 in blocking buffer containing 1% normal donkey serum. This rabbit anti-14-3-3η was made against a synthetic peptide corresponding to acetylated N-terminal sequence of sheep 14-3-3η and was used to detect 4-3-3η in mouse oocytes and eggs by Western blotting and immunofluorescence [26] and has been used effectively in other cells to detect 14-3-3η [70]. The cells were then washed in 1% blocking buffer and incubated simultaneously in DyLight™ 549 donkey anti-rabbit (711-505-152, Jackson ImmunoResearch) and FITC-conjugated goat anti-rat (711-505-152, Jackson ImmunoResearch), each diluted 1:200 in blocking buffer containing 1% donkey serum for several hours, washed again in blocking buffer, counter stained with the DNA-staining Hoechst 33342 (B 226, Sigma) at a final concentration of 1.0 μg/ml and transferred into an anti-fade solution (SlowFade®, S-2828, Invitrogen) diluted 1:1 in PBS with 0.1% PVA. All cells were imaged with the Olympus FluoView FV1000 confocal microscopy system (60X oil immersion lens with various confocal zooms; the scale bar on the images indicates the final magnification). Images were captured at multiple confocal planes. The representative images shown here are primarily images at the plane of the meiotic spindles in the cells examined.

To confirm the accumulation of 14-3-3η at meiotic spindles in mouse eggs we used an alternate primary antibody that was raised against a different immunogen. This antibody was a goat anti-14-3-3η (sc-17287, Santa Cruz Biotechnology) which was made against a peptide mapping near the C-terminus of 14-3-3η of human origin and it has been used previously to effectively detect 14-3-3η by Western blotting and immunostaining in other cell types [71]. Cells were collected, fixed, permeabilized and then incubated with the goat anti-14-3-3η and the DyLight™ 488 donkey anti-goat secondary antibody (705-485-147, Jackson ImmunoResearch). In this case, α-tubulin was stained using the rat anti-α-tubulin primary antibody (sc-69970, Santa Cruz Biotechnology) and a Texas Red-conjugated goat anti-rat secondary antibody (112-075-003, Jackson ImmunoResearch). For consistency in the presentation of the results in this paper in representing red fluorescence for 14-3-3 and green for tubulin, for this experiment the confocal image from the the DyLight™ 488 donkey anti-goat secondary antibody (green emission) was pseudocolored red and the confocal image from the Texas Red-conjugated secondary antibody (red emission) was pseudocolored green (Figure 1B,C,D). We examined 20 different egg cells and all showed the same pattern of immunofluorescent staining as described in the Results and Discussion section. For this experiment and all of the following immunocytochemical staining experiments, we processed some cells with the identical procedure, but omitted the primary antibodies (not shown). We noted minimal or no background fluorescence, indicating no nonspecific binding of the secondary antibodies when cells were imaged at the confocal settings used with the experimental cells.

### Time course assay of oocyte maturation *in vitro* to detect accumulation of 14-3-3η at meiotic spindles

Oocytes were isolated from adult (2–3 months old) CF1 mice as outlined above. All oocytes were then removed from the HEPES-buffered MEM α containing dibutyryl AMP and allowed to mature *in vitro* by incubation in bicarbonate-buffered MEM α (M4526, Sigma) containing 1X Antibiotic-Antimycotic (15240–062, Invitrogen) and 0.001 g/ml polyvinyl alcohol (P-8136, Sigma) and containing no dbcAMP. Groups of cells from the same batch of oocytes were allowed to mature *in vitro* and then were fixed at 4.5 hours, 7.5 hours, 9 hours and 12 hours. The cells were then processed for double immunofluorescence staining with rabbit anti-14-3-3η and rat anti-α-tubulin along with staining of chromosomes with Hoechst dye and imaged by confocal microscopy, as described before. At each time point, 15 different cells were examined.

### *In situ* proximity ligation assay

The Duolink *In Situ* PLA process (Olink Bioscience) allows visualization of sites of interaction between two proteins within cells *in situ*. Oocytes were collected and then maintained in bicarbonate-buffered MEM α containing 0.1 mg/ml dbcAMP in a humidified chamber with 5% carbon dioxide at 37°C. After 24 hours the media was replaced with bicarbonate-buffered MEM α without dbcAMP releasing the oocyte from meiotic arrest. At 13 hours the *in vivo* matured eggs were fixed, permeabilized and processed for *in situ* PLA using the manufacturer’s protocol and solutions modified to accommodate the standard method of manipulating eggs in media drops under oil. Cells were incubated overnight at 4°C simultaneously in rabbit anti-14-3-3η (AHP1046, AbD Serotec) and goat polyclonal antibody to human α-tubulin (03–15500, American Research Products), each diluted 1:200 in Duolink antibody diluent. Following four washes in the Duolink wash buffer A, the cells were incubated in Duolink PLUS, anti-rabbit and Duolink MINUS, anti-goat PLA probes (1:5 dilution in Duolink antibody diluent) for two hours in a humidified chamber at 37°C. Cells were then washed twice in Duolink wash buffer A, incubated with ligase-ligation solution for 30 minutes in a humidified chamber at 37°C, washed two more times in Duolink wash buffer A, incubated in polymerase-amplification solution (for rolling circle amplification of the DNA strands attached to the PLA probes) for 100 minutes in humidified chamber at 37°C, washed twice in 1X Duolink wash buffer B and once for 10 min in 0.01X Duolink wash buffer B containing Hoechst 33342 dye (1 μg/mL). The eggs were then transferred to a solution of SlowFade® (S-2828, Invitrogen) diluted 1:1 in PBS with 0.1% PVA for imaging for confocal imaging. Z-stack images of cells were collected at 2 μm intervals from one side of the spindle to the other. Images are presented here as a single slice or as a compressed Z-stack of the spindle region. The *in situ* PLA sites of interaction between 14-3-3η and α-tubulin were examined in this manner in 18 different egg cells. Seven control eggs processed were for *in situ* PLA following the identical procedure, but in the absence of the primary antibodies; these cells had no PLA reaction spots indicating that there is no rolling circle amplification in the absence of the primary antibodies.

### Microinjections

A translation-blocking morpholino oligonucleotide was designed to block translation of the mouse 14-3-3η mRNA (5’-CTGCTCTCGATCCCCCATGTCGCTC-3’, Gene Tools) and it was solubilized in sterile deionized water to prepare a 2 mM solution. The injection pipettes were beveled [72], backfilled with the injection solution and connected to a semi-quantitative injection system utilizing pneumatic pressure injection (PLI-100A Pico-Injector, Harvard Apparatus). The injection pressure and the injection duration were adjusted to match calibrated injection volumes determined by measuring the diameter and calculating the volume of the sphere of the injection solution injected into inert dimethylpolysiloxane (viscosity 12,500 cSt; DMPS12M, Sigma) which covered a drop of HEPES-buffered MEM α containing the oocytes. Ten pL (approximately 5% of the oocyte cell volume) of the morpholino solution was injected into the cytoplasm of mouse oocytes to give a final concentration of approximately 0.1 mM within each oocyte.

To show that the injection alone did not have an effect on oocyte maturation and to show that the morpholino was specifically blocking the 14-3-3η mRNA, a number of control experiments were done. Some mouse oocytes were injected with 10 pL of deionized water (the vehicle for morpholino injections). In addition, two morpholino controls were used that should have no effect on 14-3-3η mRNA. Of these, the first consisted of 10 pL of a 2 mM stock non-sense morpholino that should not bind to 14-3-3η (5’-CTCGCTGTACCCCCTAGCTCTCGTC-3’, Gene Tools; the invert of the morpholino against 14-3-3η). Also, some oocytes were injected with 10 pL of a 2 mM solution of a translation-blocking morpholino against mouse 14-3-3γ mRNA (5’-GGTCCACCATCTTCACAGGGCTGAA-3’, Gene Tools). This morpholino should not bind to 14-3-3η mRNA and serves as an additional control for the morpholino injection. All injections were performed in HEPES-buffered MEM α with 0.1 mg/ml dbcAMP.

The injected oocytes and some additional control uninjected oocytes were held in prophase I meiotic arrest for 24 hours. The oocytes were maintained in a bicarbonate-buffered MEM α containing 0.1 mg/ml dbcAMP in a humidified chamber with 5% carbon dioxide at 37°C. After 24 hours the media was replaced with bicarbonate-buffered MEM α without dbcAMP. The oocytes allowed to mature *in vitro* for 13 hours and then processed for immunofluorescence and confocal microscopy, as described before with staining for 14-3-3η, α-tubulin and DNA. Images were captured at multiple confocal planes. The representative images shown are primarily images at the plane of the meiotic spindles or DNA in the cells examined. For each injection condition, the cells were classified into three categories depending on the whether the meiotic spindle was absent, deformed or normal in appearance. Nonparametric statistical analysis was done using Fisher’s exact test. First, pairwise orthogonal comparisons were made across the four control groups; because they were not different, they were combined for further analysis. The combined control group and the experimental group were compared for the absence of a spindle versus the presence of at least some spindle microtubules, and for presence of deformed spindles versus the presence of normal spindles.

## Abbreviations

dbcAMP: Dibutyryl adenosine 3^′^,5^′^-cyclic monophosphate; eCG: Equine Chorionic Gonadotropin; FITC: Fluorescein isothiocyanate; hCG: Human Chorionic Gonadotropin; MI: Meiosis I; MII: Meiosis II; MEM: Minimal Essential Medium; MO: Morpholino Oligonucleotide; PBS: Phosphaste buffered saline; PLA: Proximity Ligation Assay; PVA: Polyvinyl Alcohol; YWHA: Tyrosine 3-monooxygenase/tryptophan 5-monooxygenase activation protein; YWHAG: Tyrosine 3-monooxygenase/tryptophan 5-monooxygenase activation protein gamma polypeptide; YWHAH: Tyrosine 3-monooxygenase/tryptophan 5-monooxygenase activation protein eta polypeptide.

## Competing interests

The authors have no competing interests.

## Authors’ contributions

SD and DK designed and conducted all practical work. Both the authors reviewed the data and combined to draft the manuscript. Both authors read and approved the final manuscript.

## References

[B1] Jones K Turning it on and off: M-phase promoting factor during meiotic maturation and fertilization Mol Hum Reprod 2004 10 1 1 5 10.1093/molehr/gah009 14665700

[B2] Mehlmann LM Stops and starts in mammalian oocytes: recent advances in understanding the regulation of meiotic arrest and oocyte maturation Reproduction 2005 130 6 791 799 10.1530/rep.1.00793 16322539

[B3] Von Stetina JR Orr-Weaver TL Developmental control of oocyte maturation and egg activation in metazoan models Cold Spring Harbor Perspect Biol 2011 3 10 a005553 10.1101/cshperspect.a005553 PMC3179337 21709181

[B4] Conti M Hsieh M Zamah AM Oh JS Novel signaling mechanisms in the ovary during oocyte maturation and ovulation Mol Cell Endocrinol 2012 356 1–2 65 73 22101318 10.1016/j.mce.2011.11.002 PMC4104635

[B5] Beall S Brenner C Segars J Oocyte maturation failure: a syndrome of bad eggs Fertil Steril 2010 94 7 2507 2513 10.1016/j.fertnstert.2010.02.037 20378111 PMC2946974

[B6] Wassarman P Fujiwara K Immunofluorescent anti-tubulin staining of spindles during meiotic maturation of mouse oocytes in vitro J Cell Sci 1978 29 FEB 171 188 75210 10.1242/jcs.29.1.171

[B7] Vogt E Kirsich-Volders M Parry J Eichenlaub-Ritter U Spindle formation, chromosome segregation and the spindle checkpoint in mammalian oocytes and susceptibility to meiotic error Mutat Res Genet Toxicol Environ Mutag 2008 651 1–2 14 29 10.1016/j.mrgentox.2007.10.015 18096427

[B8] Yin S Sun X Schatten H Sun Q Molecular insights into mechanisms regulating faithful chromosome separation in female meiosis Cell Cycle 2008 7 19 2997 3005 10.4161/cc.7.19.6809 18802407

[B9] Schatten H Sun Q Centrosome dynamics during mammalian oocyte maturation with a focus on meiotic spindle formation Mol Reprod Dev 2011 78 10–11 757 768 21887720 10.1002/mrd.21380

[B10] Jones KT Lane SIR Chromosomal, metabolic, environmental, and hormonal origins of aneuploidy in mammalian oocytes Exp Cell Res 2012 318 12 1394 1399 10.1016/j.yexcr.2012.02.012 22394508

[B11] Szollosi D Calarco P Donahue R Absence of centrioles in first and second meiotic spindles of mouse oocytes J Cell Sci 1972 11 2 521 5076360 10.1242/jcs.11.2.521

[B12] Schuh M Ellenberg J Self-organization of MTOCs replaces centrosome function during acentrosomal spindle assembly in live mouse oocytes Cell 2007 130 3 484 498 10.1016/j.cell.2007.06.025 17693257

[B13] Solc P Baran V Mayer A Bohmova T Panenkova-Havlova G Saskova A Schultz RM Motlik J Aurora kinase A drives MTOC biogenesis but does not trigger resumption of meiosis in mouse oocytes matured in vivo Biol Reprod 2012 87 4 85 85 10.1095/biolreprod.112.101014 22837479 PMC3507544

[B14] Longo F Chen D Development of cortical polarity in mouse eggs - involvement of the meiotic apparatus Dev Biol 1985 107 2 382 394 10.1016/0012-1606(85)90320-3 4038667

[B15] Brunet S Maro K Cytoskeleton and cell cycle control during meiotic maturation of the mouse oocyte: integrating time and space Reproduction 2005 130 6 801 811 10.1530/rep.1.00364 16322540

[B16] Aitken A 14-3-3 proteins: A historic overview Semin Cancer Biol 2006 16 3 162 172 10.1016/j.semcancer.2006.03.005 16678438

[B17] Morrison DK The 14-3-3 proteins: integrators of diverse signaling cues that impact cell fate and cancer development Trends Cell Biol 2009 19 1 16 23 10.1016/j.tcb.2008.10.003 19027299 PMC3073487

[B18] Mackintosh C Dynamic interactions between 14-3-3 proteins and phosphoproteins regulate diverse cellular processes Biochem J 2004 381 329 342 15167810 10.1042/BJ20031332 PMC1133837

[B19] Freeman AK Morrison DK 14-3-3 Proteins: Diverse functions in cell proliferation and cancer progression Semin Cell Dev Biol 2011 22 7 681 687 10.1016/j.semcdb.2011.08.009 21884813 PMC3221730

[B20] Meek SEM Lane WS Piwnica-Worms H Comprehensive proteomic analysis of interphase and mitotic 14-3-3-binding proteins J Biol Chem 2004 279 31 32046 32054 10.1074/jbc.M403044200 15161933

[B21] Hermeking H Benzinger A 14-3-3 Proteins in Cell Cycle Regulation Semin Cancer Biol 2006 16 3 183 192 10.1016/j.semcancer.2006.03.002 16697662

[B22] Gardino AK Yaffe MB 14-3-3 Proteins as Signaling Integration Points for Cell Cycle Control and Apoptosis Semin Cell Dev Biol 2011 22 7 688 695 10.1016/j.semcdb.2011.09.008 21945648 PMC3507455

[B23] Zhou Q Kee Y Poirier CC Jelinek C Osborne J Divi S Surcel A Will ME Eggert US Mueller-Taubenberger A Iglesias PA Cotter RJ Robinson DN 14-3-3 coordinates microtubules, rac, and myosin II to control cell mechanics and cytokinesis Curr Biol 2010 20 21 1881 1889 10.1016/j.cub.2010.09.048 20951045 PMC2975807

[B24] Robinson DN 14-3-3, an integrator of cell mechanics and cytokinesis Small GTPases 2010 1 3 165 169 10.4161/sgtp.1.3.14432 21686271 PMC3116603

[B25] Pietromonaco S Seluja G Aitken A Elias L Association of 14-3-3 proteins with centrosomes Blood Cells Mol Dis 1996 22 19 225 237 9075573 10.1006/bcmd.1996.0103

[B26] De S Marcinkiewicz JL Vijayaraghavan S Kline D Expression of 14-3-3 protein isoforms in mouse oocytes, eggs and ovarian follicular development BMC Res Notes 2012 5 57 10.1186/1756-0500-5-57 22264317 PMC3292963

[B27] Fredriksson S Gullberg M Jarvius J Olsson C Pietras K Gustafsdottir SM Ostman A Landegren U Protein detection using proximity-dependent DNA ligation assays Nat Biotechnol 2002 20 5 473 477 10.1038/nbt0502-473 11981560

[B28] Soderberg O Gullberg M Jarvius M Ridderstrale K Leuchowius K Jarvius J Wester K Hydbring P Bahram F Larsson L Landegren U Direct observation of individual endogenous protein complexes in situ by proximity ligation Nat Methods 2006 3 12 995 1000 10.1038/nmeth947 17072308

[B29] Weibrecht I Leuchowius K Clausson C Conze T Jarvius M Howell WM Kamali-Moghaddam M Soderberg O Proximity ligation assays: a recent addition to the proteomics toolbox Expert Rev Proteomics 2010 7 3 401 409 10.1586/epr.10.10 20536310

[B30] Stein P Svoboda P Schultz RM Transgenic RNAi in mouse oocytes: a simple and fast approach to study gene function Dev Biol 2003 256 1 187 193 12654301 10.1016/s0012-1606(02)00122-7

[B31] Xu Z Williams CJ Kopf GS Schultz RM Maturation-associated increase in IP3 receptor type 1: Role in conferring increased IP3 sensitivity and Ca2+ oscillatory behavior in mouse eggs Dev Biol 2003 254 2 163 171 10.1016/S0012-1606(02)00049-0 12591238

[B32] Svoboda P Long dsRNA and silent genes strike back: RNAi in mouse oocytes and early embryos Cytogenet Genome Res 2004 105 2–4 422 434 15237230 10.1159/000078215

[B33] Yu JY Deng MQ Medvedev S Yang JX Hecht NB Schultz RM Transgenic RNAi-mediated reduction of MSY2 in mouse oocytes results in reduced fertility Dev Biol 2004 268 1 195 206 10.1016/j.ydbio.2003.12.020 15031116

[B34] Knott J Kurokawa M Fissore R Schultz R Williams C Transgenic RNA interference reveals role for mouse sperm phospholipase C in triggering Ca^2+^ oscillations during fertilization Biol Reprod 2005 72 4 992 996 15601914 10.1095/biolreprod.104.036244

[B35] Summerton J Weller D Morpholino antisense oligomers: Design, preparation, and properties Antisense Nucleic Acid Drug Dev 1997 7 3 187 195 10.1089/oli.1.1997.7.187 9212909

[B36] Eisen JS Smith JC Controlling morpholino experiments: don't stop making antisense Development 2008 135 10 1735 1743 10.1242/dev.001115 18403413

[B37] Summerton J Morpholino antisense oligomers: the case for an RNase H-independent structural type Biochim Biophys Acta Gene Struct Expr 1999 1489 1 141 158 10.1016/S0167-4781(99)00150-5 10807004

[B38] Summerton JE Morpholino, siRNA, and S-DNA compared: Impact of structure and mechanism of action on off-target effects and sequence specificity Curr Top Med Chem 2007 7 7 651 660 10.2174/156802607780487740 17430206

[B39] Coonrod SA Bolling LC Wright PW Visconti PE Herr JC A morpholino phenocopy of the mouse mos mutation Genesis 2001 30 3 198 200 10.1002/gene.1065 11477708

[B40] Nasevicius A Ekker SC Effective targeted gene 'knockdown' in zebrafish Nat Genet 2000 26 2 216 220 10.1038/79951 11017081

[B41] Heasman J Kofron M Wylie C beta-catenin signaling activity dissected in the early Xenopus embryo: A novel antisense approach Dev Biol 2000 222 1 124 134 10.1006/dbio.2000.9720 10885751

[B42] Homer HA Mad2 and spindle assembly checkpoint function during meiosis I in mammalian oocytes Histol Histopathol 2006 21 8 873 886 16691540 10.14670/HH-21.873

[B43] Madgwick S Hansen DV Levasseur M Jackson PK Jones KT Mouse Emi2 is required to enter meiosis II by reestablishing cyclin B1 during interkinesis J Cell Biol 2006 174 6 791 801 10.1083/jcb.200604140 16966421 PMC2064334

[B44] Furuya M Tanaka M Teranishi T Matsumoto K Hosoi Y Saeki K Ishimoto H Minegishi K Iritani A Yoshimura Y H1foo is indispensable for meiotic maturation of the mouse oocyte J Reprod Dev 2007 53 4 895 902 10.1262/jrd.19008 17519519

[B45] Sun S Wei L Li M Lin S Xu B Liang X Kim N Schatten H Lu S Sun Q Perturbation of survivin expression affects chromosome alignment and spindle checkpoint in mouse oocyte meiotic maturation Cell Cycle 2009 8 20 3365 3372 10.4161/cc.8.20.9855 19806029

[B46] Yuan J Xu B Qi S Tong J Wei L Li M Ouyang Y Hou Y Schatten H Sun Q MAPK-activated protein kinase 2 is required for mouse meiotic spindle assembly and kinetochore-microtubule attachment PLoS One 2010 5 6 e11247 10.1371/journal.pone.0011247 20596525 PMC2893158

[B47] Ou X Li S Xu B Wang Z Quan S Li M Zhang Q Ouyang Y Schatten H Xing F Sun Q p38 alpha MAPK is a MTOC-associated protein regulating spindle assembly, spindle length and accurate chromosome segregation during mouse oocyte meiotic maturation Cell Cycle 2010 9 20 4130 4143 10.4161/cc.9.20.13389 20948319 PMC3055197

[B48] Zhang C Wang Z Quan S Huang X Tong J Ma J Guo L Wei Y Ouyang Y Hou Y Xing F Sun Q GM130, a cis-Golgi protein, regulates meiotic spindle assembly and asymmetric division in mouse oocyte Cell Cycle 2011 10 11 1861 1870 10.4161/cc.10.11.15797 21552007

[B49] Zhu J Qi S Wang Y Wang Z Ouyang Y Hou Y Schatten H Sun Q Septin1 is required for spindle assembly and chromosome congression in mouse oocytes Dev Dyn 2011 240 10 2281 2289 10.1002/dvdy.22725 21932310

[B50] Homer H McDougall A Levasseur M Yallop K Murdoch A Herbert M Mad2 prevents aneuploidy and premature proteolysis of cyclin B and securin during meiosis I in mouse oocytes Genes Dev 2005 19 2 202 207 10.1101/gad.328105 15655110 PMC545877

[B51] Sanfins A Lee G Plancha C Overstrom E Albertini D Distinctions in meiotic spindle structure and assembly during in vitro and in vivo maturation of mouse oocytes Biol Reprod 2003 69 6 2059 2067 10.1095/biolreprod.103.020537 12930715

[B52] Rubio MP Peggie M Wong BHC Morrice N MacKintosh C 14-3-3s regulate fructose-2,6-bisphosphate levels by binding to PKB-phosphorylated cardiac fructose-2,6-bisphosphate kinase/phosphatase EMBO J 2003 22 14 3514 3523 10.1093/emboj/cdg363 12853467 PMC165633

[B53] Rubio MP Geraghty KM Wong BHC Wood NT Campbell DG Morrice N Mackintosh C 14-3-3-affinity purification of over 200 human phosphoproteins reveals new links to regulation of cellular metabolism, proliferation and trafficking Biochem J 2004 379 395 408 10.1042/BJ20031797 14744259 PMC1224091

[B54] Pignocchi C Doonan JH Interaction of a 14-3-3 protein with the plant microtubule-associated protein EDE1 Ann Bot 2011 107 7 1103 1109 10.1093/aob/mcr050 21558460 PMC3091805

[B55] Chaudhri M Scarabel M Aitken A Mammalian and yeast 14-3-3 isoforms form distinct patterns of dimers in vivo Biochem Biophys Res Commun 2003 300 3 679 685 10.1016/S0006-291X(02)02902-9 12507503

[B56] Jones DH Ley S Aitken A Isoforms of 14-3-3-protein can form homodimers and heterodimers in-vivo and in-vitro - implications for function as adapter proteins FEBS Lett 1995 368 1 55 58 10.1016/0014-5793(95)00598-4 7615088

[B57] Aitken A Post-translational modification of 14-3-3 isoforms and regulation of cellular function Semin Cell Dev Biol 2011 22 7 673 680 10.1016/j.semcdb.2011.08.003 21864699

[B58] Lee CG Park GY Han YK Lee JH Chun SH Park HY Kim KH Kim EG Choi Y-J Yang K Lee CW Roles of 14-3-3 eta in mitotic progression and its potential use as a therapeutic target for cancers Oncogene 2013 32 1560 1569 10.1038/onc.2012.170 22562251

[B59] Kumagai A Dunphy W Binding of 14-3-3 proteins and nuclear export control the intracellular localization of the mitotic inducer Cdc25 Genes Dev 1999 13 9 1067 1072 10.1101/gad.13.9.1067 10323858 PMC316939

[B60] Conklin DS Galaktionov K Beach D 14-3-3-proteins associate with cdc25-phosphatases Proc Natl Acad Sci USA 1995 92 17 7892 7896 10.1073/pnas.92.17.7892 7644510 PMC41252

[B61] Uchida S Kuma A Ohtsubo M Shimura M Hirata M Nakagama H Matsunaga T Ishizaka Y Yamashita K Binding of 14-3-3 beta but not 14-3-3 sigma controls the cytoplasmic localization of CDC25B: binding site preferences of 14-3-3 subtypes and the subcellular localization of CDC25B J Cell Sci 2004 117 14 3011 3020 10.1242/jcs.01086 15173315

[B62] Giles N Forrest A Gabrielli B 14-3-3 acts as an intramolecular bridge to regulate cdc25B localization and activity RID A-6597-2008 J Biol Chem 2003 278 31 28580 28587 10.1074/jbc.M304027200 12764136

[B63] Zhang Y Zhang Z Xu X Li X Yu M Yu A Zong Z Yu B Protein Kinase A modulates Cdc25B activity during meiotic resumption of mouse oocytes Dev Dyn 2008 237 12 3777 3786 10.1002/dvdy.21799 19035343

[B64] Pirino G Wescott MP Donovan PJ Protein kinase A regulates resumption of meiosis by phosphorylation of Cdc25B in mammalian oocytes Cell Cycle 2009 8 4 665 670 10.4161/cc.8.4.7846 19223768

[B65] Evans JP Robinson DN The spatial and mechanical challenges of female meiosis Mol Reprod Dev 2011 78 10–11 769 777 21774026 10.1002/mrd.21358 PMC3196790

[B66] Douglas ME Davies T Joseph N Mishima M Aurora B and 14-3-3 coordinately regulate clustering of centralspindlin during cytokinesis Curr Biol 2010 20 10 927 933 10.1016/j.cub.2010.03.055 20451386 PMC3348768

[B67] Ding J Swain JE Smith GD Aurora Kinase-A regulates microtubule organizing center (MTOC) localization, chromosome dynamics, and histone-H3 phosphorylation in mouse oocytes Mol Reprod Dev 2011 78 2 80 90 10.1002/mrd.21272 21274965

[B68] Yang K Li S Chang C Tang CC Lin Y Lee S Tang TK Aurora-C kinase deficiency causes cytokinesis failure in meiosis I and production of large polyploid oocytes in mice Mol Biol Cell 2010 21 14 2371 2383 10.1091/mbc.E10-02-0170 20484572 PMC2903667

[B69] Snow AJ Puri P Acker-Palmer A Bouwmeester T Vijayaraghavan S Kline D Phosphorylation-dependent interaction of tyrosine 3-monooxygenase/tryptophan 5-monooxygenase activation protein (YWHA) with PAD16 following oocyte maturation in mice Biol Reprod 2008 79 2 337 347 10.1095/biolreprod.108.069328 18463355 PMC2575841

[B70] Martin H Rostas J Patel Y Aitken A Subcellular-localization of 14-3-3-isoforms in rat-brain using specific antibodies J Neurochem 1994 63 6 2259 2265 7964746 10.1046/j.1471-4159.1994.63062259.x

[B71] Titus MA Tan J Gregory CW Ford OH Subramanian RR Fu H Wilson EM Mohler JL French FS 14-3-3 eta amplifies androgen receptor actions in prostate cancer Clin Cancer Res 2009 15 24 7571 7581 10.1158/1078-0432.CCR-08-1976 19996220 PMC2795092

[B72] Kline D Carroll DJ Quantitative microinjection of mouse oocytes and eggs Microinjection: Methods and Applications, Volume 518 Methods in Molecular Biology 2009 New York: Humana Press, Inc 135 156 10.1007/978-1-59745-202-1_11 19085140

