# Correction: evidence for the requirement of 14-3-3eta (YWHAH) in meiotic spindle assembly during mouse oocyte maturation

**DOI:** 10.1186/1471-213X-14-20

**Published:** 2014-05-23

**Authors:** Santanu De, Douglas Kline

**Affiliations:** 1 Department of Biological Sciences, Kent State University, Kent, OH 44242, USA

## Correction

After publication of this work [1] we determined that the one of the experiments used an incorrect combination of primary and secondary antibodies. In the Results and Discussion and in the Methods we referred to an experiment which used a goat anti-14-3-3η antibody. In combination with this antibody, a secondary antibody used in the experiment was incorrect. It would, in fact be labeling another secondary in addition to the primary antibody it was intended to label. Therefore, we are not confident of this experiment and would like to remove the section from the results. The intent was only to reaffirm a previous finding. This particular experiment is not critical and does not alter the basic findings or final conclusions of the paper.

In light of this mistake we would ask the reader to disregard the last part of the first paragraph of the Results and Discussion section, beginning with the statement, “In the present study…”. Also, reference to this experiment in the Methods section can also be omitted (the paragraph beginning with: “To confirm …” and the sentences through “…as described in the Results and Discussion section.”). Images in the accompanying Figure 1A-D should be omitted and for clarity we have relabeled the figure as shown below (Figure 1) emphasizing the localization 14-3-3η in the meiotic spindle using the correct combination of a rabbit antibody to 14-3-3η and the correct fluorescent secondary antibody.

**Figure 1:**
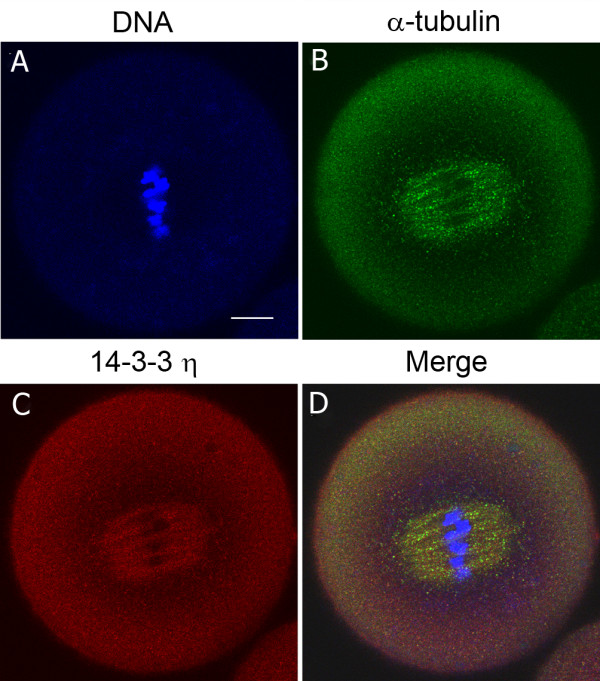
**The 14-3-3η protein accumulates at the metaphase II spindle of the mouse egg matured *in vitro*.** Cells were fixed, permeabilized and immunolabeled for confocal double immunofluorescence using a primary antibody against the 14-3-3η protein (red), an antibody to α-tubulin (green) and counterstained with Hoechst 33342 (blue) to visualize DNA. **(A-D)** A representative in vitro-matured egg cell that was held in prophase I arrest for 24 hours, released from the arrest and examined at 13 hours with a rabbit antibody recognizing the N-terminal end of the 14-3-3η protein **(C)**. The merged image **(D)** is an overlay of immunofluorescence images from the three channels. Scale bar represents 10 μm.
